# Discipline in Stages: Regulating CD8^+^ Resident Memory T Cells

**DOI:** 10.3389/fimmu.2020.624199

**Published:** 2021-03-19

**Authors:** Rut Mora-Buch, Shannon K. Bromley

**Affiliations:** Center for Immunology and Inflammatory Diseases, Division of Rheumatology, Allergy and Immunology, Massachusetts General Hospital, Harvard Medical School, Boston, MA, United States

**Keywords:** tissue resident memory T cell, T cell differentiation, recall response, microenvironment, transcriptional regulation

## Abstract

Resident memory CD8^+^ T (T_RM_) cells are a lymphocyte lineage distinct from circulating memory CD8^+^ T cells. T_RM_ lodge within peripheral tissues and secondary lymphoid organs where they provide rapid, local protection from pathogens and control tumor growth. However, dysregulation of CD8^+^ T_RM_ formation and/or activation may contribute to the pathogenesis of autoimmune diseases. Intrinsic mechanisms, including transcriptional networks and inhibitory checkpoint receptors control T_RM_ differentiation and response. Additionally, extrinsic stimuli such as cytokines, cognate antigen, fatty acids, and damage signals regulate T_RM_ formation, maintenance, and expansion. In this review, we will summarize knowledge of CD8^+^ T_RM_ generation and highlight mechanisms that regulate the persistence and responses of heterogeneous T_RM_ populations in different tissues and distinct microenvironments.

## Introduction

**Figure 1:**
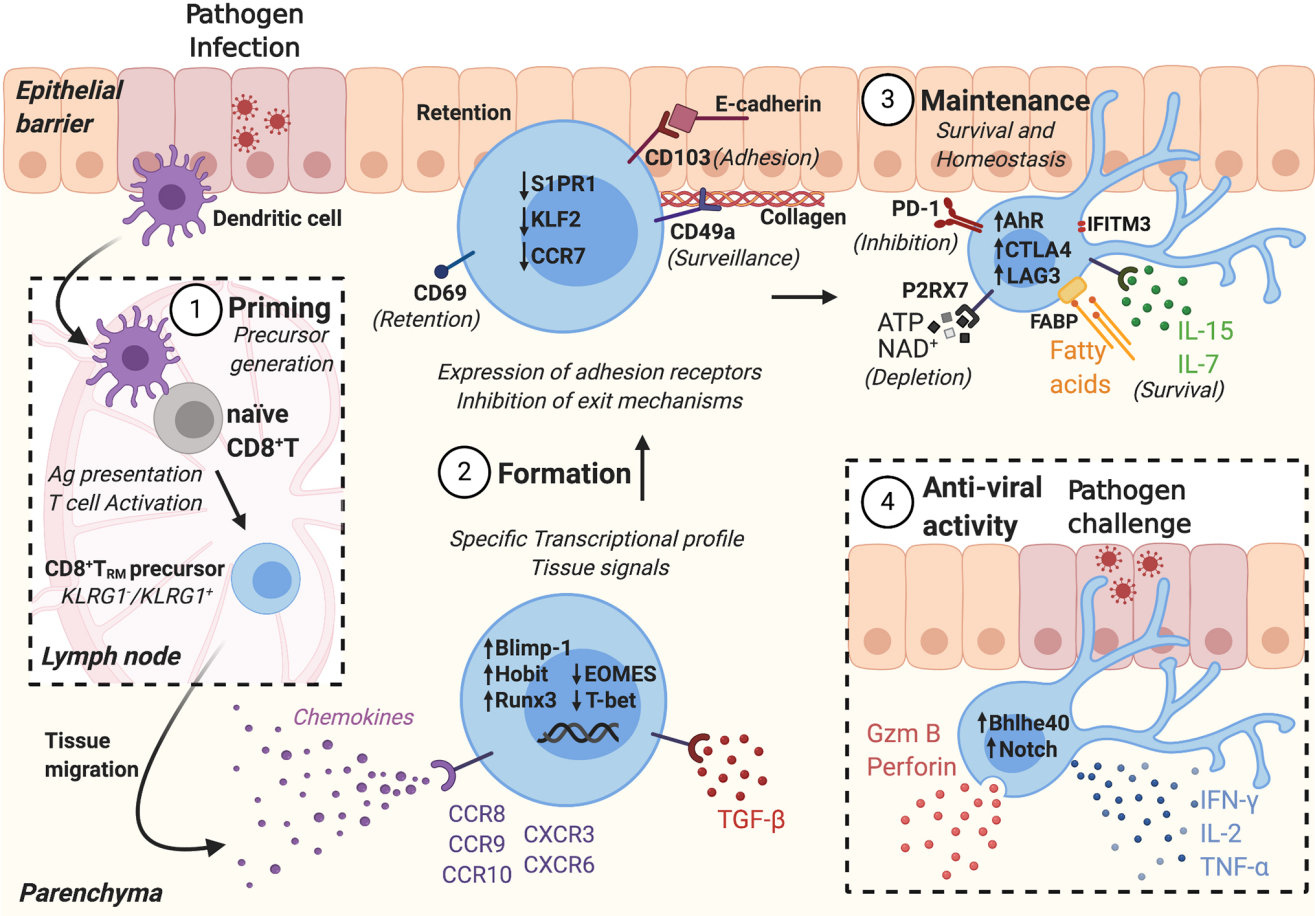
CD8^+^ T_RM_ formation and anti-viral activity is tightly regulated in different stages. 1) Following pathogen infection, tissue dendritic cells (DCs) migrate to the draining lymph nodes and present antigens to naïve T cells. Antigen-specific naïve T cells are activated, generating CD8^+^ T_RM_ precursors. 2) CD8^+^ T_RM_ precursors migrate into peripheral tissues, following chemotactic signals. CD8^+^ T_RM_ formation depends on tissue signals that activate a T_RM_ transcriptional profile, including the expression of adhesion receptors and inhibition of exit mechanisms. 3) CD8^+^ T_RM_ are maintained in the tissue where they receive survival signals and express inhibitory receptors to maintain tissue homeostasis. 4) During secondary infection, CD8^+^ T_RM_ are activated, secrete effector molecules, and amplify the immune response.

Long-term memory to pathogens is a key feature of the adaptive immune system. The ability of memory T cells to mount rapid and potent responses against previously encountered antigens maintains human health by controlling infections and tumor growth; it also provides the rationale for designing vaccines against pathogens and immune therapies to treat cancer. By recirculating through blood and lymph, circulating memory T cells may provide broad tissue immune surveillance. However, recent findings demonstrated that long after the resolution of infection, the majority of memory CD8^+^ T cells are non-circulating (1). Rather, most CD8^+^ memory T cells are stably maintained in tissues as tissue resident memory T cells (T_RM_) that exhibit transcriptional and phenotypic characteristics distinct from circulating memory CD8^+^ T cells (2). Early studies identified T_RM_ within the epithelial compartment of barrier tissues including skin, lung, and intestine (3–8). Later, T_RM_ were identified in the tissue stroma as well as in non-barrier tissues such as liver, brain, and secondary lymphoid organs including spleen and lymph nodes (LN) (9–12). CD8^+^ T_RM_ deliver highly effective, localized responses to pathogen challenge (4, 8). Additionally, CD8^+^ T cells with a T_RM_ phenotype are a target candidate for anti-tumor immunotherapy (13–15) and predict an improved prognosis in several different cancers (16–23). Although T_RM_ provide potent protection against pathogens and tumors, T_RM_ dysregulation has been linked to immune-mediated diseases including psoriasis (24), vitiligo (24), and alopecia areata in the skin (25), and inflammatory bowel disease in the intestine (26). Additionally, T_RM_ develop following sensitization to allergens and play a role in hypersensitivity reactions in allergic contact dermatitis (27, 28) and asthma (29). Finally, T_RM_ have been linked to fixed drug eruptions (30), as well as rejection of solid organ transplants (31). This review will discuss intrinsic and extrinsic mechanisms that promote CD8^+^ T_RM_ formation, maintenance and function for defense against invading pathogens, as well as mechanisms that limit T_RM_ formation and effector response to prevent excessive inflammation and tissue damage (**Figure 1**).

## Stage 1: Priming and Precursor Formation: CD8^+^ T Cells, Born or Trained to be T_RM_?

Following cognate antigen recognition, naïve CD8^+^ T cells become activated, proliferate and give rise to heterogeneous progeny with distinct effector and memory cell fates. Recent experimental evidence suggests that extrinsic signals can influence CD8^+^ T cell fate even before antigen recognition (32) (**Box 1**). After antigen activation, the majority of activated T cells die by apoptosis during the contraction phase of the immune response, but a small minority survive to become memory CD8^+^ T cells. Whether activated T cells survive may depend on external signals, including growth factor availability, antigen, and inflammation, as well as internal signals such as transcription factor and growth factor receptor expression. Multiple, non-mutually exclusive models have been proposed to explain the development of diverse populations of effector and memory CD8^+^ T cells (34). For example, the fixed lineage model proposes that commitment to effector or memory T cell lineages occurs soon after T cell stimulation, as early as the first cell division and may result from the asymmetric division of effector fate-associated factors. On the other hand, the decreasing potential model posits that early effector cells have memory potential that is lost with increased or prolonged stimulation with antigen or cytokines. More recently, Rosato et al., have proposed an expanded model of decreasing potential to include CD8^+^ T_RM_. They propose that the differentiation of CD8^+^ T cells along a continuous axis of decreasing memory potential is irreversible. However, they also divide cells based on parallel paths of migration status-stationary or migratory, that may be altered by extrinsic stimuli including TCR signaling and inflammation (35), reflecting the cells’ plasticity.

Box 1 **Pre-Programmed Naïve CD8^+^ T Cells: The Existence of a Stage 0.**

Although current models suggest that a single naïve T cell has the potential to differentiate into all effector and memory subsets depending on the antigen, costimulatory, and cytokine stimulation they receive, recent experimental evidence suggests that extrinsic signals influence CD8^+^ T cell fate even before antigen recognition. Recent work by Mani et al. demonstrated that extrinsic cytokine signaling can imprint naïve CD8^+^ T cells for subsequent T_RM_ formation. Migratory DCs expressing TGF-β-activating integrins in the LN activate TGF-β and epigenetically condition naïve CD8^+^ T cells, even before antigen stimulation, to form epithelial CD8^+^ T_RM_ in the skin (32). These results suggest that during immune homeostasis, the LN environment affects future T cell fate. In addition, research using a tamoxifen-inducible fate-mapping mouse model to mark CD8^+^ T cells made in the thymus during fetal, neonatal, and adult stages, Smith et al. demonstrated that naïve CD8^+^ T cells generated during different developmental stages, fetal vs. adult, acquire different phenotypes upon antigen encounter. These results suggest that CD8^+^ T cell fate may be controlled by the timing of naïve precursor cell maturation in the thymus (33). These studies open the possibility of additional regulatory mechanisms and signals that impact future CD8^+^ T_RM_ generation even before inflammatory or antigen insult. Future studies are needed to better understand how intrinsic and extrinsic signals during naïve CD8^+^ T cell generation and homeostasis influence CD8^+^ T cell fate.

### CD8^+^ T_RM_ Precursor Differentiation

Expression of KLRG1 and CD127 has been used to define the memory potential of effector CD8^+^ T cells around the peak of the immune response. Adoptive transfer studies suggest that KLRG1^+^ CD127^−^ short-lived effector cells (SLEC) tend to die following clearance of antigen, whereas KLRG1^−^ CD127^+^ memory precursor effector cells (MPEC) preferentially survive to give rise to memory CD8^+^ T cells (36). Using a single cell adoptive transfer approach, Stemberger et al. tracked the progeny of individual naïve CD8^+^ T cells. Using CD62L and CD127 as phenotypic markers, and IL-2, TNF-α, IFN-γ and CD107a expression as functional readouts, they demonstrated that diverse effector and memory CD8^+^ T cells can arise from the same naïve precursor T cell (37). Additionally, single cell tracing experiments using adoptive transfer of barcode labeled OT-I T cells and systemic or local infection models, confirmed that both effector and memory CD8^+^ T cell subsets derive from the same precursors in the naïve T cell pool (38). Moreover, TCR repertoire analysis of antigen-activated CD8^+^ T cells demonstrated that 35 days post-immunization, CD8^+^ memory T cells recovered from the skin share a common clonal origin with memory CD8^+^ T cells isolated from draining and distant LNs, suggesting that T_RM_ and circulating memory T cells can develop from an individual naïve T cell (39). Together, these results suggest that memory T cell fate is not imprinted on naïve T cells, but rather that individual naïve T cells can give rise to all effector and memory CD8^+^ T cell subsets. However, recent data suggest that although the majority of naïve T cells contribute to both circulating memory and CD69^+^ CD103^+^ T_RM_ cell populations, the contribution of individual clones to each memory pool varies (40). Additionally, analysis of individual T cell families (a naïve T cell and its progeny) demonstrated that clonal expansion and differentiation of T cells bearing the same TCR are heterogeneous, and so the contribution of the progeny of individual naïve T cells varies between primary versus recall responses (41).

Substantial effort has focused on identifying CD8^+^ T_RM_ precursor cells and defining when CD8^+^ T cells commit to a T_RM_ fate (**Supplementary Table 1**). Like circulating memory CD8^+^ T cells, CD8^+^ T_RM_ can also differentiate from KLRG1^−^ precursor cells. Mackay et al. demonstrated that KLRG1^−^, but not KLRG1^+^, HSV-specific gBT-I effector T cells sorted from the spleens of mice 6 days post-HSV infection, generated cutaneous CD103^+^ T_RM_ cells following their adoptive transfer into HSV-infected recipient mice (42). Subsequent studies suggested that CD8^+^ T_RM_ are derived from MPEC after their entry into peripheral tissues. For example, following infection with *Listeria monocytogenes* (LM), splenic MPEC and SLEC lack expression of the T_RM_ receptors, CD69 and CD103. However, MPEC but not SLEC recovered from the intestine express CD103 and CD69 (43). Additionally, elegant work performed by Kurd et al. used single-cell RNA sequencing to define the gene expression patterns of individual CD8^+^ T cells in the spleen and small intestine intraepithelial lymphocyte (siIEL) compartments over the course of lymphocytic choriomeningitis virus (LCMV) infection. Four days post-infection, the earliest time-point that virus specific CD8^+^ T cells are detected within intestinal tissue, activated CD44^hi^ small intestinal CD8^+^ T cells display a transcriptional profile distinct from splenic CD44^hi^ CD8^+^ T cells. Even at day 3 following infection, splenic CD8^+^ T cells do not resemble siIEL, suggesting that circulating precursors are not committed to a T_RM_ fate until after entry into the tissue (44). In contrast, using lineage tracing and single-cell transcriptome analysis, Kok et al. identified a subset of circulating effector CD8^+^ T cells at the peak of effector T cell expansion after skin DNA vaccination that are enriched for T_RM_ fate-associated gene expression and have a higher propensity to form T_RM_ (40). Because the clonal composition of T_RM_ recovered from anatomically separate skin immunization sites is similar, they proposed that a committed T_RM_ precursor pool exists in the circulation, before entry into the tissue. Although the nature, timing or location of the early signals that imprint the ability to form T_RM_ before tissue entry were not defined by this study, work by Mani et al. suggests that during immune homeostasis, naïve CD8^+^ T cells are epigenetically preconditioned for T_RM_ formation through their interaction with migratory dendritic cells (DCs) expressing TGF-β-activating integrins (32).

Recent studies suggest that effector cells may maintain plasticity to dedifferentiate and seed the memory pool. Using a KLRG1^Cre^ reporter system that allows tracking of KLRG1^+^ T cells over time, Herndler-Brandstetter et al. demonstrated that early post infection, KLRG1^+^ effector CD8^+^ T cells can downregulate KLRG1 and differentiate into all memory T cell lineages, including CD8^+^ T_RM_ in the lung, intestine, and skin, and mediate effective protective immunity (45). Additionally, work by Youngblood et al. examined the transcriptional and epigenetic changes in naïve CD8^+^ T cells during differentiation to effector and memory cells over the course of an acute LCMV infection. Whole genome bisulfite sequencing analysis demonstrated that epigenetic repression of naïve-associated genes in effector CD8^+^ T cells can be reversed in cells that develop into long-lived memory CD8^+^ T cells, while key effector genes including *Gzmb* and *Prf1* remain demethylated (46). These studies suggest that effector CD8^+^ T cells may not have a fixed fate and contribute to the diversity of the memory T cell pool.

### Intrinsic Control of CD8^+^ T_RM_ Precursor Generation: TCR Affinity and Signal Strength

The finding that CD8^+^ T_RM_ and circulating memory CD8^+^ T cells can express identical TCR sequences (37) counters the hypothesis that TCR affinity or signal strength determines CD8^+^ T_RM_ differentiation. However, intrinsic signals, including TCR signal strength and antigen affinity can influence CD8^+^ memory T cell development. For example, a study using OT-I TCR transgenic mice with a point mutation in the conserved antigen receptor transmembrane (CART) motif suggests that effector and memory T cell differentiation require different signals. Both WT and mutant T cells differentiate comparably into effector T cells. However, mutant cells fail to polarize TCR to the immunological synapse, have decreased NFKB induction, and this impaired TCR signaling is correlated with decreased memory CD8^+^ T cell differentiation (47). Additionally, studies have demonstrated that higher affinity TCR interactions direct CD8^+^ T cells to a CD62L^−^ T_EM_ fate, whereas lower TCR affinities promote CD62L^+^ T_CM_ formation (48). Several studies also support the idea that TCR affinity and signal strength have a direct and unique impact on CD8^+^ T_RM_ formation. For example, in a mouse model of persistent polyomavirus (MPyV) infection, high-affinity CD8^+^ CD69^+^ T_RM_ cells in the brain originate from high-affinity CD62L^−^ effector cells present in the tissue during acute infection (49). In contrast, in a separate study again using a model of MPyV, the data instead suggested that lower TCR stimulation strength improves memory potential and generates functional brain CD62L^−^ CD69^+^ T_RM_ cells (50). Similarly, in an acute influenza infection model, lower affinity TCR stimulation is more likely than higher affinity interactions to induce T_RM_ formation, suggesting that TCR affinity can influence T_RM_ differentiation (51) and may provide a mechanism to regulate the diversity of antigen-specific T_RM_ within tissues.

Additional intrinsic CD8^+^ T cell characteristics may also affect CD8^+^ T cell fate. For example, variation in expression levels of signaling proteins including CD8, ERK-1 and SHP-1 generates a range of CD8^+^ T cell responsiveness to antigen stimulation. However, co-regulation of signaling proteins limits this variability, potentially providing a mechanism to diversify cell fate, but control self-reactivity (52). Similarly, Marchingo et al. used a high-throughput clonal assay to simultaneously measure the expansion fate of multiple clonal families expressing identical TCR in a single culture well. Their results demonstrate that following stimulation, progeny from clonal families stop dividing and return to quiescence at or near the same generation, suggesting that regulation of CD8^+^ T cell expansion fate is at the level of the individual clone (53). Stochastic variation in costimulatory and cytokine receptor expression by naïve CD8^+^ T cells, for example differences in CD28 receptor expression, influences the generation at which an initial individual activated cell reverts to a quiescent state (53). Future *in vivo* research is required to determine whether stochastic variation in protein expression by naïve T cells, either before or during early priming, has an effect on subsequent T cell fate, including CD8^+^ T_RM_ differentiation.

### Extrinsic Control of CD8^+^ T_RM_ Precursor Generation

#### Antigen and Antigen Presentation During Priming

Contact between DCs and antigen-specific CD8^+^ T cells can influence the fate of responding T cells (54–57). DCs carrying pathogen-derived antigens migrate to draining LN and prime naïve CD8^+^ T cells. The interaction between DCs and T cells within the LN occurs in three stages initiated by brief encounters, followed by more stable contacts and concludes with a return to brief contacts and rapid T cell migration, accompanied by the commencement of T cell proliferation (58). Multiphoton intravital microscopy (MP-IVM) allowed for the analysis of how and when the interactions between naïve CD8^+^ T cells and DCs determine effector and memory CD8^+^ T cell differentiation, and suggested that stable contacts and a high antigen concentration are critical to induce memory T cell generation (59). Additionally, Ballesteros-Tato et al. showed that more abundant influenza epitopes are preferentially cross-presented at late times in the primary response, and responding T cells are favorably programmed toward a memory cell fate (60). More recently, studies have identified specific cross-priming DC populations that favor CD8^+^ T_RM_ precursor differentiation. In a mouse model of vaccinia virus (VACV) infection, DNGR-1^+^ Batf3-dependent DCs prime naïve CD8^+^ T cells within the LN to form T_RM_ within skin or lung (61). Further, human studies and experiments using a humanized mouse metastatic lung model identified a subset of activated CD88^−^CD1c^+^CD163^+^CD14^+/−^ DCs, or DC3s, that prime naïve CD8^+^ T cells and induce TGF-β-triggered CD103 expression (62).

#### Route of Entry and Inflammatory Milieu

The gene expression profile and half-life of activated CD8^+^ T cells are determined by many signals during pathogen invasion, such as antigen presentation by mature DCs, T cell stimulation by receptor ligands and inflammatory cytokines (63). During T cell priming, different LN environments direct expression of distinct T cell homing receptors (5, 64, 65). For example, oral, but not intranasal mouse infection with LM induces efficient homing and precursor development of CD8^+^ T_RM_ in the intestinal epithelium (43). In contrast, CD8^+^ T cells lodge within the skin following infection with herpes simplex virus (HSV) *via* either skin scarification or subcutaneous injection after controlling for priming efficiency (66).

Distinct patterns of cytokine expression within the LN environment during priming also modulate precursor formation and program CD8^+^ T cell fate (67, 68). For instance, IL-12 produced during LCMV infection induces T-bet expression in CD8^+^ T cells in a dose-dependent manner, and favors the development of SLEC over MPEC (69, 70). On the other hand, IL-10 plasma levels early following immunization with peptide antigen and adjuvant strongly correlates with the frequencies of antigen specific T_RM_ in the lung of mice and non-human primates at a memory time point. Production of IL-10 by monocytes acts in an autocrine manner to release TGF-β during priming, increasing CD8^+^ T cell responsiveness to subsequent TGF-β stimulation, and thereby favors the formation of CD8^+^ CD103^+^ T_RM_ (71).

## Stage 2: Mechanisms That Encourage CD8^+^ T_RM_ to Settle in Peripheral Tissues

### CD8^+^ T_RM_ Phenotype and Transcriptional Regulation

Following CD8^+^ T cell activation and clonal expansion within draining LN, T_RM_ precursors migrate to non-lymphoid tissues. Entry into peripheral tissues induces a unique T_RM_ phenotype that promotes CD8^+^ T cell retention and prevents egress (**Supplementary Table 2**). More than a decade ago, Masopust et al. demonstrated that as early as 7 days following intestinal LCMV infection, the gut microenvironment induces a unique CD8^+^ T cell differentiation program; CD8^+^ IELs express both CD69 and CD103, while splenic circulating memory CD8^+^ T cells do not (72). Similarly, Ray and colleagues found that within 8 days following influenza infection, flu-specific CD8^+^ T cells recovered from the lung were predominantly CD49a^+^, while those recovered from the mediastinal LN were CD49a^−^ (7). This phenotype persisted at memory timepoints. More recently, Mackay et al. performed microarray analysis of CD103^+^ CD8^+^ T_RM_ isolated from the skin, gut, and lungs of mice and determined that CD8^+^ T_RM_ express a unique T_RM_ transcriptional signature that is distinct from circulating memory CD8^+^ T cells. This analysis identified 37 transcripts commonly regulated by T_RM_ from all three tissues, including *S1pr1*, *Itga1* and *Itgae*, encoding sphingosine 1-phosphate receptor-1 (S1P1), CD49a and CD103, respectively (42). A similar human CD8^+^ T_RM_ core transcriptional profile was also later defined (73, 74).

CD69 is perhaps the most ubiquitous marker for CD8^+^ T_RM_ cells in mouse and human tissues (74, 75). CD69 forms a complex with the chemoattractant receptor S1P1, inducing S1P1 internalization and thereby impairing S1P-directed lymphocyte exit *via* afferent lymphatic vessels (42, 75, 76). In parallel, downregulation of kruppel-like factor 2 (KLF2), the transcription factor that drives S1P1 gene expression, is necessary for the establishment of CD8^+^ T_RM_ in tissues (77, 78). CD69 expression by CD8^+^ T cells is necessary for the generation of CD8^+^ T_RM_ in the kidney (79) and skin (75). However, recent work demonstrated that CD69 expression is dispensable for the formation of CD8^+^ T_RM_ in small intestine, lung, and female reproductive tract (79). Like CD69, the integrin, CD103 has also been used extensively as a marker for CD8^+^ T_RM_. CD103 is expressed by CD8^+^ T_RM_ in the epithelial compartment of multiple tissues (4, 42, 80, 81) and is thought to mediate T_RM_ retention through its interaction with e-cadherin. However, although CD103 is necessary for CD8^+^ T_RM_ accumulation within epithelium, it is dispensable for T_RM_ persistence in other tissue compartments (42, 43). For instance, Bergsbaken et al. demonstrated that following *Yersinia pseudotuberculosis* (Yptb) infection, a CD103^−^ CD8^+^ T_RM_ cell population persists long-term in the intestinal lamina propria (82). Additionally, CD49a, the α chain of integrin α1β1, is expressed by CD8^+^ T_RM_ and promotes their accumulation within multiple mouse and human tissues (4, 7, 24, 74, 83, 84).

Comparison of CD8^+^ T_RM_ and circulating memory CD8^+^ T cells transcriptomes has identified several transcription factors that are differentially expressed between memory CD8^+^ T cells subsets. Expression of *Zfp683*, encoding homolog of Blimp1 in T cells (Hobit) is upregulated in CD8^+^ T_RM_ and is necessary for CD8^+^ T_RM_ cell development in the skin, gut, liver and kidney of mice (83). Interestingly, Hobit has been described in several other cell lineages, including CD4^+^ T, Natural killer (NK), NKT, and Mucosal-associated invariant T (MAIT) cells, and acts as a transcriptional regulator of residency (83, 85–87). Hobit, together with the transcription factor Blimp1 coregulate genes required for tissue egress (83). In the absence of Hobit and Blimp1, Klf2, S1p1, and CCR7 are de-repressed. However, although human lung and liver CD69^+^ CD8^+^ T cells express Hobit, so do human circulating CD45RA^+^ CD27^−^ and CD45RA^−^CD27^−^ CD8^+^ T cells, suggesting that Hobit may not specifically promote human CD8^+^ T_RM_ differentiation (88). Additionally, the requirement of Hobit for T_RM_ differentiation may be tissue-specific. In the lung, Blimp1, but not Hobit, is required for the formation of virus-specific CD8^+^ T_RM_ in a mouse influenza infection model (89). Moreover, Milner et al. used single-cell RNA sequencing (scRNA-seq) analysis to characterize CD8^+^ siIEL populations over time following LCMV infection. They demonstrated heterogeneity in the CD8^+^ siIEL T_RM_ and identified distinct resident memory CD8^+^ T cell populations based on their expression of the transcription factors Blimp1 and Id3. Previous studies demonstrated that Blimp1^hi^ expression favors an effector T cell fate (90). Accordingly, Milner et al. showed that compared to Blimp1^lo^ Id3^hi^ siIEL, Blimp1^hi^ Id3^lo^ siIEL CD8^+^ T cells dominate the early response and express increased effector-associated genes. Nonetheless, lower numbers of Blimp1^hi^ Id3^lo^ siIEL CD8^+^ T cells are still present in the tissue at memory timepoints. Although Blimp1 was expressed by a subset of CD8^+^ T cells across multiple non-lymphoid tissues, expression of Id3 was more restricted, raising the possibility that T_RM_ transcriptional programs may be regulated by the local tissue microenvironment (91).

Two T-box transcription factors, Eomesodermin (Eomes) and T-bet, control CD8^+^ CD103^+^ T_RM_ cell formation in lung, skin, and brain. Although T_CM_ express both Eomes and T-bet (92), expression of these transcription factors must be downregulated for CD8^+^ T_RM_ development. While extinguishment of Eomes expression is required for CD8^+^ CD103^+^ T_RM_ cell formation (93, 94), residual T-bet expression maintains CD8^+^ T cell IL-15Rβ expression and IL-15 responsiveness for long-term T_RM_ survival within lung and skin (94, 95). Additionally, recent data generated using ATAC-seq and transcriptional profiling identified the transcription factor, Runx3 as a central regulator of CD8^+^ T_RM_ differentiation (32, 44, 73, 96). Runx3, previously described as a transcriptional regulator of CD8^+^ effector T cells (97), promotes expression of tissue residency genes while suppressing genes involved in tissue egress. Runx3^−/−^ CD8^+^ T cells have elevated T-bet levels, suggesting that Runx3 represses T-bet expression; knockdown of T-bet expression in Runx3^−/−^ CD8^+^ T cells increases CD8^+^ T_RM_ numbers and restores CD69 and CD103 expression. Runx3 deficiency results in loss of CD8^+^ T_RM_ in barrier (skin and lung) as well as non-barrier (salivary gland and kidney) tissues, suggesting that Runx3 may regulate CD8^+^ T_RM_ formation independent of the local tissue milieu (96).

CD8^+^ T_RM_ generation and long-term maintenance are also regulated by nuclear receptor subfamily 4 group A member 1 (NR4A1) (44, 98). Nr4a1, also known as Nur77, is rapidly induced following TCR stimulation and regulates CD8^+^ T cell proliferation and effector function (99). In a mouse model of influenza infection, similar numbers of co-adoptively transferred *Nr4a1* ^−/−^ and wild-type antigen-specific CD8^+^ T_RM_ are recovered at the effector phase. However, fewer *Nr4a1* ^−/−^ CD8^+^ T cells are recovered from the liver and intestine at a memory time point, although similar numbers are recovered from lung (98). Finally, scRNA-seq analysis of siIEL and splenic CD8^+^ T cells over the course of LCMV infection demonstrated increased expression of *Nr4a2*, Junb proto-oncogene (*Junb*) and FOS-like 2 (*Fosl2*) in siIEL relative to splenic CD8^+^ T cells. Knockdown of these genes results in impaired formation of siIEL CD8^+^ T_RM_ compared to circulating memory CD8^+^ T cells, although the mechanisms were not determined (44).

### *In Situ* Antigen Dependence

Following vesicular stomatitis virus (VSV) infection, local antigen presentation is required to drive CD103 expression by infiltrating CD8^+^ T cells that promotes their persistence within brain (9). Similarly, local antigen recognition is required for T_RM_ formation in the lung (100, 101). Following influenza infection, viral antigen-bearing pulmonary monocytes interact with influenza-specific CD8^+^ T cells *in vivo* and can induce CD103 expression by CD8^+^ T cells *in vitro* (102). While localized inflammation can recruit CD8^+^ T cells into the lung, in the absence of local antigen recognition, memory CD8^+^ T cells fail to express the retention receptors CD69, CD103, and CD49a or persist long-term (103). However, the requirement of antigen recognition within peripheral tissues for CD8^+^ T_RM_ formation is not absolute. CD8^+^ CD103^+^ T_RM_ can be generated in the absence of antigen recognition in barrier tissues, including skin, intestine, and female reproductive tract (104–106). Nonetheless, subsequent studies demonstrated that local recognition of antigen dramatically increases the formation of CD8^+^ T_RM_ in VACV-infected skin (107, 108). Moreover, local competition between CD8^+^ T cells of different specificities for different viral epitopes shapes the repertoire of cutaneous CD8^+^ T_RM_ cells following VACV infection (107), underlining the importance of local antigen recognition in regulating the establishment of CD8^+^ T_RM._

### Tissue-Derived Signals: Cytokines, Inflammatory Molecules, and Other Immune Cells Signals

The local tissue cytokine microenvironment influences CD8^+^ T_RM_ phenotype. TGF-β is critical for the formation of CD103^+^ CD8^+^ T_RM_ in several tissues, including the siIEL compartment, skin epidermis, lung, and kidney (105, 109–111). CD8^+^ T cells expressing mutant TGF-β receptors fail to express CD103 or persist within multiple peripheral tissues (42, 43, 81, 105, 109). Recent data suggest that epidermal CD8^+^ T_RM_ cells require transactivation of autocrine TGF-β for their long-term persistence, and competition for limited TGF-β influences which clones persist within the epidermis (112). CD8^+^ T cell TGF-β responsiveness is controlled by the transcription factors EOMES and T-bet, and downregulation of Eomes and T-bet is required for CD8^+^ T cell TGF-β responsiveness and CD8^+^ T_RM_ formation (94). Additionally, recent research has identified a role for the transcriptional cofactor, SKI, in regulating CD8^+^ T cell CD103 expression. Using an LCMV infection model, Wu et al. demonstrated that ectopic expression of SKI proto-oncogene restricts CD103 expression by CD8^+^ T cells *in vitro* and *in vivo*. SKI is recruited to the *Itgae* locus to suppress CD103 transcription by preventing histone acetylation in a Smad4-dependent manner. Moreover, in the absence of Smad4, CD103 is constitutively expressed by CD8^+^ T cells even in the absence of TGF-β signaling, suggesting that modulation of TGF-β-SKI-Smad4 pathway could determine CD8^+^ CD103^+^ T_RM_ generation (111).

Inflammatory cytokines produced in response to local infection, and the chemokines they induce also regulate T_RM_ formation and phenotype. IFN-γ and the IFN-γ-induced chemokines, CXCL9 and CXCL10 have been shown to orchestrate CD8^+^ T_RM_ precursor migration and localization within tissues in multiple infection models. For example, following influenza infection, IFN-γ produced by CD4^+^ T cells promotes the localization of CD8^+^ T cells to the airways, thereby controlling their exposure to TGF-β (95). Similarly, following genital HSV-2 infection, IFN-γ induces local expression of the CXCR3 ligands, CXCL9 and CXCL10 that promotes CD8^+^ T cell localization and long-term persistence within the tissue (113). Furthermore, local application of these chemokines is sufficient to recruit CD8^+^ T cells into the genital tract where they are retained long-term and enhance memory response to reinfection (106). Similarly, keratinocytes express CXCL9 and CXCL10 during HSV skin infection. KLRG1^−^ CD8^+^ T_RM_ precursors show preferential migration to these chemokines *ex vivo* compared to KLRG1^+^ effector CD8^+^ T cells. Moreover, following intradermal injection, CXCR3^−/−^ CD8^+^ T cells generate fewer CD103^+^ T_RM_ than adoptively transferred WT CD8^+^ T cells, suggesting that CXCR3 mediates T_RM_ precursor entry into the epidermis where locally activated TGF-β may promote subsequent epidermal CD8^+^ CD103^+^ T_RM_ generation (42, 114). Additionally, CXCR3-directed localization of type I Treg expressing the TGF-β activating integrin, αvβ8, within local inflammatory sites promotes CD8^+^ T_RM_ generation in the intestine, liver, and lung. Positioning of these Treg adjacent to effector CD8^+^ T cells promotes CD8^+^ T_RM_ generation *via* activated TGF-β availability (115). In contrast, generation of CD8^+^ CD103^−^ T_RM_ following oral Yptb infection is independent of TGF-β signaling, but requires CXCR3-dependent clustering of effector CD8^+^ T cells with CXCL10-producing CX3CR1^+^ intestinal cells in areas of inflammation within the intestinal lamina propria, suggesting that the microenvironment formed by immune cell aggregates supports CD8^+^ T_RM_ formation (116). Indeed, IL-12 and IFN-β produced by intestinal macrophages during Yptb infection prevents TGF-β-induced CD103 expression by CD8^+^ T cells, favoring the differentiation of CD8^+^ CD103^−^ T_RM_ cells (82). Thus, inflammatory cytokines not only function to induce local chemokine expression to promote the recruitment of T_RM_ precursors into tissues, but also influence the differentiation of CD8^+^ T cells within the tissue, providing a mechanism to promote T_RM_ phenotypic diversity.

Several additional chemokine receptors may also participate in the formation of CD8^+^ T_RM_ within peripheral tissues. For example, expression of the intestinal homing chemokine receptor CCR9 by CD8^+^ siIEL is increased compared to their circulating counterparts throughout their differentiation (5, 44). Additionally, expression of CXCR6 and CCR10 by mouse CD8^+^ T cells are required for optimal CD8^+^ T_RM_ formation in the skin (117). Although CD8^+^ T_RM_ formation in mouse skin appears to be CCR8-independent (117), human cutaneous CD69^+^ CD103^+^ T_RM_ express CCR8, raising the possibility that CCR8 and its ligands may regulate human cutaneous CD8^+^ T_RM_ generation or function (118, 119).

Competition for survival cytokines may also impact CD8^+^ T_RM_ accumulation within tissues. A recent report using an LCMV infection model demonstrated that NK1.1^+^ innate lymphoid cells (ILCs) control the accumulation of memory CD8^+^ T cells in salivary glands. Specifically, establishment of CD8^+^ T_RM_ is enhanced in anti-NK1.1^+^ antibody pretreated mice. The authors propose that ILCs might compete for survival signals such as IL-7, although no specific mechanism was determined (120). Similarly, following HSV skin infection, CD8^+^ T_RM_ formation is accompanied by a concomitant local decrease in dendritic epidermal γδ T cells, suggesting possible competition for survival cytokines within the epidermal niche.

Costimulatory signals also play a role in the establishment of CD8^+^ T_RM_ within tissues. During influenza infection, Zhou et al. showed that interaction of the costimulatory molecule, 4-1BB with its ligand 4-1BBL is necessary for the induction of long-lived lung-resident CD103^+^ and CD103^−^ memory CD8^+^ T cell populations (121). In addition, glucocorticoid-induced TNFR-related protein ligand (GITRL), expressed by lung monocyte-derived inflammatory antigen presenting cells, provides a costimulatory signal for lung CD8^+^ T cells expressing GITR during influenza infection. GITRL/GITR interaction in the LN and lung is required for the differentiation of CD8^+^ T_RM_ precursors and the formation of CD8^+^ T_RM_ within the lung parenchyma (122).

Additional microenvironmental cues may also regulate the generation of CD8^+^ T_RM_. For example, microRNA-155 is upregulated during infection in response to TLR signaling and inflammatory cytokines (123). CD8^+^ T_RM_ are established in the brain following infection of mice with neuroinvasive LM, and their accumulation is decreased in the absence of miR-155 (124). Also, CD8^+^ T cells require P2RX7 expression for CD8^+^ T_RM_ formation in the siIEL, female reproductive tract, kidney, salivary glands, and liver. Extracellular ATP is released during inflammation and injury, and is sensed by the purinergic receptor, P2RX7. Upon CD8^+^ T cell activation, expression of TGF-β receptors is transiently down-regulated. Extracellular ATP derived from intestinal microbiota, activated cells and/or damaged tissue restores TGF-βRII expression and TGF-β responsiveness, resulting in CD8^+^ T cell CD103 upregulation, KLF2 downregulation, enhanced mitochondrial function and T_RM_ formation (125). On the other hand, microbiota depletion by antibiotic treatment increases the antigen load following LM infection and promotes CXCR3-directed CD8^+^ T cell accumulation within the large intestinal lamina propria, resulting in increased mucosal CD8^+^ T_RM_ accumulation and response (126).

## Stage 3: CD8^+^ T_RM_ Maintenance in Peripheral Tissues

### *In Situ* Antigen Dependence

CD8^+^ T_RM_ persist long-term within several tissues, including intestinal IEL (105), vaginal mucosa (106), and skin (104, 127) independent of cognate antigen recognition. In contrast, lung CD8^+^ T_RM_ are rapidly lost from the tissue. Several studies suggest that cognate antigen recognition is required for the persistence of lung CD8^+^ T_RM_. Residual local antigen persistence may promote continuous development of lung T_RM_ and allow for the maintenance of CD8^+^ T_RM_ within the tissue (128). Following influenza infection, CD8^+^ T_RM_ receive chronic local TCR stimulation even weeks after the clearance of infectious influenza virus. Furthermore, tamoxifen-inducible H-2D^b^ depletion or B7-CD28 blockade starting at least three weeks post-infection results in impaired maintenance of CD8^+^ T_RM_ cells within the lung (129). Based on these findings, novel methods are being developed in attempt to prolong the persistence of CD8^+^ T_RM_ within the lung. Combined subcutaneous and intranasal vaccination of mice with an adenovirus vector expressing influenza antigen is reported to induce persistent antigen expression in the lungs and maintains T_RM_ within the lung for at least one year post-vaccination (130). Continual recruitment of circulating CD8^+^ T_EM_ may convert into T_RM_ following antigen recognition and help to sustain T_RM_ within the interstitium.

However, the requirement of circulating memory CD8^+^ T cell recruitment for the long-term maintenance of lung CD8^+^ T_RM_ has been questioned by a recent study using parabiosis and intravascular staining to exclude analysis of CD8^+^ T cells within the circulation. Takamura et al. demonstrated that CD8^+^ T_RM_ can be retained in specific niches created at sites of tissue regeneration within the lung parenchyma, distant from lymph vessels, and independent of CD8^+^ T cell recruitment from the circulation (100). Still, the half-life of CD8^+^ T_RM_ within lung airways is less than 14 days (131), and so they propose that maintenance of airway memory CD8^+^ T cells may require residual antigen-driven reactivation of CD8^+^ T_RM_ in the lung parenchyma and recruitment into the airways (100, 132). More recently, an additional mechanism has been proposed to maintain regional immune memory specific for lung pathogens. Stolley et al. demonstrated that following influenza infection, CD8^+^ T cells migrate to draining mediastinal LN *via* lymphatic vessels. These cells express CD103 and CD69, are maintained long-term within the LN in an antigen-independent manner and maintain effector molecule expression. As such, repositioning and persistence of CD8^+^ T_RM_ within the draining mediastinal LN may provide a means to maintain regional immune memory despite rapid attrition of lung CD8^+^ T_RM_ (133).

### CD8^+^ T_RM_ Receptors and Transcriptional Regulators

Maintenance of CD8^+^ T_RM_ is thought to require expression of retention receptors that act as adhesive anchors (Formation markers and transcriptional regulators in stage 2, **Supplementary Table 2**, and **Supplementary Table 3**). CD103 binds to E-cadherin, which is expressed in skin epidermis (134) and intestinal epithelium (5, 105). This interaction is thought to anchor CD8^+^ T_RM_ within the epithelial compartment of tissues and facilitate their long-term residence (135). Similarly, CD49a binds collagen type I and IV, and also facilitates CD8^+^ T_RM_ persistence within skin, lung, and intestine (7, 84, 136). In addition to its adhesive function, CD49a may also provide a pro-survival signal, limiting CD8^+^ memory T cell apoptosis (7).

Although CD69 is required for CD8^+^ T_RM_ establishment in several tissues, it may not be required for their long-term maintenance. Following mouse influenza infection, CD8^+^ T_RM_ are retained long-term within the lung independent of CD69 expression. Early after infection, CD69 is important for the accumulation of CD8^+^ T cells within the airways to inhibit strong S1P1-mediated exit signals. However, once CD8^+^ T_RM_ are established, CD69 is dispensable even though the cells maintain residual S1P1 reactivity (100). Downregulation of KLF2, the transcription factor that drives S1P1 expression, may preclude the need for continued CD69 expression in T_RM_ to inhibit any S1P-mediated exit signal. Moreover, physical separation of T_RM_ from lymphatic vessels by their positioning within lung niches or within the epidermis may also facilitate their retention within tissues independent of CD69.

The expression patterns of several transcription factors that regulate CD8^+^ T_RM_ formation are maintained long-term in established T_RM_ (Transcriptional regulators in stage 2, **Supplementary Table 2** and **Supplementary Table 3**). However, Milner et al. found divergent transcription factor expression patterns in CD8^+^ T cells with distinct phenotypic properties during different stages of T_RM_ formation and maintenance. Specifically, while Blimp1^hi^ Id3^lo^ siIEL CD8^+^ T cells are abundant at the effector phase of the immune response, Blimp1^lo^ Id3^hi^ siIEL CD8^+^ T cells progressively accumulate over time, and are more abundant at the memory phase of the response. Moreover Blimp1^lo^ Id3^hi^ siIEL CD8^+^ T cells have higher recall proliferative capacity and multipotency than Blimp1^hi^ siIEL CD8^+^ T cells (91). Additionally, Aryl hydrocarbon receptor (AhR) also regulates CD8^+^ T_RM_ maintenance. Expression of AhR is increased in skin CD8^+^ T_RM_ compared to naïve or circulating memory T cells. While *Ahr* ^−/−^ CD8^+^ T cells initially enter into sites of DNFB-induced skin inflammation, over time, they disappear from the skin but not spleen (134), suggesting that AhR is required for the long-term persistence of cutaneous CD8^+^ T_RM_. Accordingly, AhR expression is increased in mouse intestinal T_RM_ compared to circulating memory CD8^+^ T cells following LCMV infection (44), as well as in human lung CD8^+^ CD103^+^ T_RM_ compared to circulating memory T cells (73). Finally, Notch signaling regulates the maintenance of CD8^+^ CD103^+^ T_RM_ in the lung by regulating both CD103 expression and CD8^+^ T_RM_ metabolism (73).

### Tissue-Derived Signals: Cytokines, Inflammatory Molecules, and Other Immune Signals.

TGF-β is not only required for the establishment of CD8^+^ T_RM_ in multiple barrier tissues, but also to preserve their phenotype and long-term persistence in the intestine (109). Similarly, after cutaneous CD103^+^ CD8^+^ T_RM_ have been established, neutralization of the TGF-β-activating integrin, αvβ6, results in reduced numbers of T_RM_ in the epidermis but not LN or spleen over time (114). These results suggest that continuous TGF-β signaling is required for the long-term persistence of epidermal CD8^+^ T_RM_.

Survival cytokines also provide for the long-term sustenance of tissue-resident CD8^+^ T cells. Both IL-7 and IL-15 are required for the persistence of CD8^+^ T_RM_ in the skin (94, 137). In contrast, maintenance of T_RM_ in the lung and intestine is IL-15-independent (138, 139). On the other hand, IL-12 regulates Bcl-2 expression to promote the survival of CD8^+^ CD103^−^ T_RM_ within the intestinal lamina propria (82).

Although P2RX7 promotes CD8^+^ T_RM_ formation within the intestine (125), Stark et al. demonstrated that sterile tissue damage led to loss of established WT, but not *P2rx7* ^−/−^ CD8^+^ T_RM_ from the liver (140). They found that TCR triggering downregulates P2RX7 expression, and so proposed that tissue damage-induced depletion of established T_RM_ might free space for the formation of new CD8^+^ T_RM_ with infection-relevant specificities. In contrast, Wakim et al. determined that persistent expression of the anti-viral transmembrane protein, IFITM3 by lung CD103^+^ CD8^+^ T cells promotes the survival and maintenance of CD8^+^ T_RM_ at sites of viral infection. Following influenza infection, cognate antigen induces persistent IFITM3 expression preferentially by lung CD8^+^ T_RM_ compared to splenic memory CD8^+^ T cells. CD8^+^ T_RM_ that lack IFITM3 expression exhibit increased susceptibility to influenza infection compared to IFITM3^+^ CD8^+^ T_RM_, and are selectively lost following virus challenge (141).

Finally, CD8^+^ T_RM_ long-term survival and protective function require lipid uptake and oxidative metabolism. Fatty-acid-binding proteins 4 and 5 (FABP4 and FABP5) are required for the long-term maintenance of CD8^+^ T_RM_ within the skin following VACV infection, and for CD8^+^ T_RM_-mediated protection from viral challenge (142). However, CD8^+^ T_RM_ exhibit distinct patterns of FABP gene expression depending on their tissue of residence. An additional study demonstrated that following HSV infection, skin CD8^+^ T_RM_ express *Fabp4* and *Fabp5*, but lack expression of other FABP isoforms. However, following LCMV infection, liver CD8^+^ T_RM_ highly express *Fabp1*, some *Fabp4*, but no *Fabp5*. In contrast, siIEL CD8^+^ T_RM_ express *Fabp1*, *Fabp2*, and *Fabp6*, but negligible *Fabp4* and *Fabp5*. These differences in FABP expression are determined by tissue-derived signals, and by altering FABP expression, CD8^+^ T cells can adapt to different host tissues (143).

## Stage 4: Pathogen Challenge

### Location and Relocation

CD8^+^ T_RM_ are positioned to provide a first line of host defense in response to pathogen challenge. Recognition of cognate antigen stimulates CD8^+^ T_RM_ to rapidly secrete cytokines that induce expression of anti-viral and anti-bacterial genes, activate innate immune cells, and enhance chemokine and adhesion receptor expression for increased recruitment of circulating immune cells (144–146). Following tissue entry, circulating memory CD8^+^ T cells can undergo antigen-dependent CD69^+^ CD103^−^ T_RM_ differentiation (147) as well as antigen-independent CD69^+/−^ CD103^+/−^ T_RM_ differentiation (148, 149) *in situ*. Additionally, intravital microscopy studies revealed that established CD8^+^ T_RM_ proliferate within the female reproductive tract and skin upon cognate antigen encounter. These cells dominate the recall response and contribute more than circulating memory CD8^+^ T cells to the pool of secondary T_RM_ cells (148, 149).

At homeostasis, CD8^+^ T_RM_ persist long-term within peripheral tissues, separate from the circulation. However, following antigen reencounter, CD8^+^ T_RM_ exhibit plasticity. Beura et al. determined that CD8^+^ CD69^+^ T_RM_ in the draining LNs derive from cells present in the upstream nonlymphoid tissue (11). Complementary studies by Behr et al. used Hobit reporter mice to demonstrate that CD69^lo^ Hobit^+^ antigen specific T cells accumulate in the draining LNs in the effector phase after reinfection, and upregulate CD69 expression in the secondary memory phase, forming LN T_RM_. Virus challenge not only induces local proliferation of CD8^+^ T_RM_ cells in peripheral tissues that can participate in the accumulation of secondary T_RM_ in the draining LN, but also, formation of circulating memory CD8^+^ T cells downstream of CD8^+^ T_RM_. Studies using Hobit lineage tracer mice revealed that Hobit^+^ CD8^+^ T_RM_ can downregulate Hobit expression upon antigen encounter and form KLRG1^+^ CXC3CR1^+^ circulating T_EM_ with enhanced capacity to protect against reinfection (150). Similarly, Fonesca et al. demonstrated that following challenge, small intestinal iEL T_RM_ give rise to circulating T_CM_ and T_EM_. These ex-T_RM_ cells are epigenetically poised for migration back to the tissue of origin and T_RM_ re-differentiation (151).

### CD8^+^ T_RM_ Antigen Reencounter: Dependence on CD11c^+^ DCs

Intravital confocal microscopy illustrated that CD8^+^ T_RM_ actively patrol skin epithelium in search of cognate antigen, raising the possibility that T_RM_ within barrier tissues do not depend on antigen delivery by professional APCs (152). In line with this hypothesis, Masopust et al. demonstrated that following depletion of ~90% of host DC in CD11c-DTR bone marrow chimeric mice, T_RM_ still proliferate in response to challenge with cognate peptide antigen (149). In contrast, in the vaginal mucosa, T_RM_ reactivation following HSV-2 challenge depends on CD301b^+^ DCs (153). In addition, transplantation of the dorsal root ganglia of HSV-infected mice under the kidney capsule of naive mice induces viral reactivation. Here, the CD8^+^ T_RM_ proliferative response is initiated by recruitment of CD11b^+^ CD11c^+^ DCs. Together, these results suggest that the DC requirement for CD8^+^ T_RM_ response to antigen challenge may be context dependent. Indeed, in models of LCMV and influenza infection, cDCs are dispensable for lung CD8^+^ T_RM_ reactivation. Rather either hematopoietic or non-hematopoietic antigen presenting cells are sufficient, but they induce different T_RM_ functional outputs. Whereas antigen presentation by hematopoietic cells reduces gene transcription of chemokines and cytokines such as *Ccl1*, *Ccl3*, *Ccl9*, and *Ifng*, activation by nonhematopoietic cells promote transcription of genes involved in cell cycle and proliferation but curbs type I interferon stimulated genes (154).

### Patrolling the Tissue: Surveillance and Motility

Although T_RM_ remain resident long-term in peripheral tissues, they are not sessile cells; T_RM_ continuously patrol the local area for invading pathogens. Upon cognate antigen recognition, CD8^+^ T_RM_ become rounded and arrest their migration before undergoing proliferation *in situ* (148, 149). However, intravital microscopy studies demonstrated that depending on their tissue of residence, T_RM_ display different migration speeds and morphologies. T_RM_ migrate within skin epidermis, albeit slowly at a rate of ~1.3 µm/min, and extend dendrites laterally to probe their surroundings for cognate antigen (134). Imaging of the mouse uterus after acute LCMV infection revealed that CD8^+^ T_RM_ migrate at different rates within the stroma of the female reproductive tract and this migratory speed correlates with collagen density. T_RM_ within the collagen-rich perimetrium migrate more slowly than in the less collagen-rich myometrium where T_RM_ exhibit motility rates that are similar to those of circulating lymphocytes in LNs (149). Interestingly, a recent study in influenza-infected mice suggests that the collagen receptor, CD49a promotes CD8^+^ T cell motility within the trachea to facilitate tissue surveillance (155). In contrast, CD103 restrains T_RM_ motility in both trachea and skin (117, 155). How changes in the local microenvironment following challenge with distinct pathogens might affect CD8^+^ T_RM_ phenotype and migratory behavior requires additional study.

### Antiviral Activity: Effector Molecule Expression

CD8^+^ T_RM_ provide immediate effector functions against secondary infections (**Supplementary Table 4**). The transcriptional profiles of both mouse and human CD8^+^ T_RM_ exhibit higher expression of effector molecules compared to circulating memory CD8^+^ T cells (73, 74, 93, 105, 156). Constitutive expression of mRNAs encoding effector molecules may facilitate rapid T_RM_ response. For example, notch signaling contributes to the maintenance of constitutive *Ifng* expression by lung T_RM_ (73). Notch signaling transactivates *Ifng*, increasing *Ifng* expression by T_RM_ independent of TCR stimulation. Following recognition of cognate antigen, CD8^+^ T_RM_ secrete IFN-γ, IL-2 and TFN-α, inducing a rapid recall response at the site of pathogen invasion (146, 156–158). IFN-γ induces vascular cell adhesion molecule 1 (VCAM-1) expression by endothelial cells, as well as production of inflammatory chemokines that recruit circulating immune cells, resulting in amplification of the memory response (146). Additionally, resting lung CD8^+^ T_RM_ constitutively express *CCL3*, *CCL4*, C*CL20* and *XCL1* (73), and intestinal CD8^+^ T_RM_ express *Ccl3* and *Ccl4* (44), suggesting that CD8^+^ T_RM_ themselves express genes to rapidly amplify the memory immune response.

CD8^+^ T_RM_ targeted secretion of the cytotoxic proteins, perforin and granzyme B, destroy target cells. While circulating memory CD8^+^ T cells lack cytotoxic protein expression, T_RM_ that form within intestinal IEL, liver, and brain following LCMV infection express granzyme B during quiescence (72, 156, 159). Constitutive expression of granzyme B might promote rapid control of pathogen infection. In contrast, airway CD8^+^ T_RM_ are reported to be poorly cytolytic, even in the presence of antigen stimulation (157). The nutrient-poor airway environment induces cellular stress, limiting T_RM_ effector function and survival at homeostasis, and perhaps providing a mechanism to prevent unnecessary epithelial damage (160).

### Controlling T_RM_ Activity: Inhibitory Molecules and Metabolic Arrest

Inhibitory molecule expression may be critical to prevent T_RM_-mediated damage in barrier tissues. The inhibitory surface protein programmed death protein 1 (PD-1), upregulated by exhausted T cells and tumor infiltrating lymphocytes (TILs), is also expressed by CD8^+^ T_RM_ in mouse and human tissues (74, 161). Multiple studies suggest that PD-1 may provide T_RM_ functional restraint. For example, PD-1 expression by T cells correlates with response to anti-PD-1 blockade treatment in patients with cancer (162). Additionally, CD8^+^ PD-1^hi^ T_RM_ cells in human pancreas may maintain immune homeostasis through interactions with resident macrophages; in samples from chronic pancreatitis, CD8^+^ T cells exhibit reduced PD-1 expression (163). Moreover, following influenza infection, antigen specific CD8^+^ T cells in the lung acquire both a memory and exhausted phenotype, including PD-1 surface expression. Blocking PD-1 ligand (PD-L1) promotes exhausted-like T_RM_ cell expansion, and augments T_RM_ cell function, enhancing T_RM_-mediated protection from reinfection. However, anti-PD-L1 treatment also causes chronic tissue fibrotic sequelae, suggesting that inhibitory receptors are important for balancing immune protection and fibrotic processes (129). Similarly, CD8^+^ T_RM_ that form in the epidermis following acute contact hypersensitivity reaction express inhibitory checkpoint receptors that limit T_RM_ reactivation. Treatment with inhibitory molecule antagonists increases the magnitude and severity of eczema exacerbations (27).

Human lung CD8^+^ T_RM_ express not only PD-1, but also genes encoding inhibitory molecules such as CTLA4, BTLA, LAG3, SPRY1, and the adenosine receptor A2AR (73). Similarly, a recent study using sc-RNA seq demonstrated that inhibitory receptors including *Ctla4*, *Lag3*, *Cd101*, and *Tigit*, are upregulated early during formation of intestinal IEL CD8^+^ T cells in an acute LCMV infection model, suggesting a possible role in T_RM_ differentiation (44). Moreover, following influenza infection, differences in T_RM_ inhibitory molecule expression are observed depending on the T cell epitope, suggesting that initial TCR-MHCp interactions may determine not only T cell activation, but also inhibitory programs (161).

The balance between CD8^+^ T_RM_-mediated immune response and immune pathology may also be regulated by alterations in mitochondrial membrane composition. CD8^+^ T_RM_ express early activation markers, contain cytolytic proteins, and have the capacity to release cytokines. However, epithelial T_RM_ are metabolically arrested in a semi-activated state. Alterations in the mitochondrial membrane, including the cardiolipin composition, regulate IEL proliferation, and effector functions (164).

Finally, CD8^+^ T_RM_ adaptation to the environment is regulated by mitochondrial gene expression. The transcription factor, Bhlhe40 is highly expressed in mouse and human CD8^+^ T_RM_ compared to circulating memory CD8^+^ T cells (44, 165), and promotes T_RM_ mitochondrial gene expression. Bhlhe40^−/−^ CD8^+^ T_RM_ exhibit decreased oxygen consumption and enhanced mitochondrial damage. Additionally, Bhlhe40 deficiency results in reduced acetyl-CoA and histone acetylation of T_RM_ effector loci. Lack of Bhlhe40 reduces the production of IFN-γ, granzyme B and TNF by CD8^+^ T_RM_, suggesting that Bhlhe40 promotes epigenetic programs permissive for effector gene expression. PD-1 signaling inhibits Bhlhe40 expression. Importantly, however, targeting downstream epigenetic machinery rescues CD8^+^ T_RM_ mitochondrial function and cytokine production in the absence of Bhlhe40, suggesting a possible mechanism for improved immunotherapy (165).

## Discussion

Over the last decade, scientists around the globe have contributed to the study of CD8^+^ T_RM_. Rapid progress has been achieved in understanding the generation, regulation, and protective or pathogenic functions of T cells that reside within tissues. Since the discovery of CD8^+^ T_RM_, much effort has focused on elucidating the transcriptional networks and mechanisms that regulate these cells. These studies have identified core transcriptional signatures for both mouse and human CD8^+^ T_RM_ that promote their long-term retention and maintenance. However, with increasing data examining T_RM_ formation and function in multiple tissues and infection models, it has become increasingly clear that T_RM_ are a heterogeneous pool of cells with plastic properties. T_RM_ formation and phenotype are influenced by extrinsic signals such as antigen, cytokines, nutrients, costimulatory, and inhibitory signals within the LN and tissue microenvironments, as well as by intrinsic receptor and signaling protein expression. These factors can shape T_RM_ differentiation, maintenance and response, and their variability in different tissues and inflammatory settings promotes T_RM_ diversity between organs, and even within the same tissue. Although a great deal has already been learned, an improved understanding of the mechanisms that regulate T_RM_ formation and/or function in varied tissue environments is necessary not only to prevent autoimmune diseases, but also to improve cancer treatments and vaccine strategies.

## Author Contributions

RM-B drafted and edited the manuscript and figures. SB edited and approved the final version of the manuscript. All authors contributed to the article and approved the submitted version.

## Funding

This work was supported by NIH grant R01 AI121546 (SB) and by a National Eczema Association Catalyst Research Grant NEA19-CRG121 (RM-B).

## Conflict of Interest

The authors declare that the research was conducted in the absence of any commercial or financial relationships that could be construed as a potential conflict of interest.
