# Supplementary material for: Discipline in Stages: Regulating CD8+ Resident Memory T Cells
Source: Front Immunol. 2021 Mar 19;11:624199. doi: 10.3389/fimmu.2020.624199 (PMC8017121; doi:10.3389/fimmu.2020.624199)
Supplement: Supplementary file 1 [file Table_1.pdf]

Supplementary Table 1. Stage 1: Priming and CD8$^+$ T_{RM} precursor formation.

**Stage1: Priming and precursor development**			**Tissue**	**Model**	**References**
TCR Affinity	Low		Lung	Influenza	(50)
			Brain	Polyomavirus	(48)
	High		Brain	Polyomavirus	(49)
Antigen presentation	DCs cross-priming		Skin	VV	(60)
			Lung	VV	(60)
				Humanized tumor model	(61)
Route of infection	Dependent	Oral	SI IEL	LM	(42)
Cytokines	IL-12	Inhibit	Spleen	LCMV, LM	(69)
	IL-10	Induce	Lung	a-CD40ab-PolyIC-HIVenvpeptides	(70)

Table abbreviations: LM: listeria monocytogenes; LCMV: lymphocytic choriomeningitis virus; VV: vaccinia virus; SI: Small intestine; IEL: Intraepithelial lymphocytes.
