# Supplementary material for: Discipline in Stages: Regulating CD8+ Resident Memory T Cells
Source: Front Immunol. 2021 Mar 19;11:624199. doi: 10.3389/fimmu.2020.624199 (PMC8017121; doi:10.3389/fimmu.2020.624199)
Supplement: Supplementary file 2 [file Table_2.pdf]

Supplementary Table 2. Stage 2: CD8^{+} T_{RM} formation in peripheral tissues.

Stage 2: Formation in the tissue			Tissue	Model	References
TRM markers	CD69	+	SI IEL, LN	LCMV	(71,75)
			Skin	HSV	(41,74,82)
			Lung, intestine	Healthy human tissue	(73)
			Kidney	LCMV, influenza	(78)
		-	SI IEL, lung, FRT	LCMV, influenza, VSV	(78)
	CD103	+	SI IEL	LCMV	(71)
			Skin	HSV	(4,41)
			FRT	HSV	(79)
			Liver	LCMV	(82)
			Lung	Influenza	(80,154)
				Human	(72)
		-	Intestinal LP	LM	(42)
				Yptb	(81)
			Liver	LCMV	(82)
	CD49a	+	Lung	Healthy human tissue	(73)
				Influenza	(7,154)
			Intestine	Healthy human tissue	(73)
			Skin	Human vitiligo, psoriasis	(24)
				HSV	(4,82,83)

	S1p1 ↓	Skin	HSV	(41,74)
		Intestine	LCMV	(43)
Transcriptional regulators	KLF2↓	Intestine	LCMV	(43,77)
		SG, kidney, brain,	LCMV	(77)
	Hobit↑	Skin	HSV	(82)
		Liver, intestine, kidney	LCMV	(82)
	Blimp↑	Skin	HSV	(82)
		Intestine, liver, kidney, lung	LCMV	(82,88)
		Intestinal IEL	LCMV	(90)
	NR4A1↑	Liver, Lung, intestine	LCMV	(43,97)
	NR4A2↑	Intestine	LCMV	(90)
	Junb↑	Intestine	LCMV	(43)
	Eomes↓	Brain	LM	(92)
		Skin	HSV	(93)
	T-bet↓	Skin	HSV	(93)
		Lung	Influenza	(94)
	Runx3↑	Intestine	LCMV	(43)
		Skin	HSV	(32)
		Intestine	LCMV, LM	(90)
		Lung	Human	(72)

Antigen	Dependent	Brain	VSV, LM	(9)
		Lung	Influenza	(99–102,160)
	Independent	Skin	DNFB-HSV	(103)
			MVA	(106)
			VV	(107)
		Intestinal IEL	LCMV	(104)
Tissue migration	CXCR3-CXCL9/CXCL10	Skin	HSV	(41)
		Intestinal LP	Yptb	(115)
		Intestine	LM	(125)
		FRT	HSV	(105)
	CCR9	Intestinal IEL	LCMV	(5,43)
	CCR8	Skin	Human	(117,118)
	CCR10-CCL27	Skin	HSV	(116)
	CXCR6	Skin	DNFB	(116)
Cytokines	TGF-β	Intestine	LCMV	(104,108)
		Skin	HSV	(41)
		Kidney	LCMV	(109)
		Lung	Influenza	(94)
			LCMV	(110)
	IL-12	Intestinal LP	Yptb	(81)
	IFN-β	Intestinal LP	Yptb	(81)
Immune cells	$CD4^+$ Induce	Lung	Influenza	(94)

			Intestinal LP	Yptb	(115)
	NK1.1+ ILC	Inhibit	Salivary gland	LCMV	(119)
Immune mediators	4-1BB-4-1BBL		Lung	Influenza	(120)
	GITRL		Lung	Influenza	(121)
	miR-155		Brain	LM	(123)
	ATP/ P2RX7		Intestine	LCMV	(169)

Table abbreviations: + Expressed; - Not expressed; ↑ upregulated; ↓ downregulated; DNFB: 2,4-Dinitro-1-fluorobenzene; MVA: poxvirus modified Vaccinia Ankara; LM: listeria monocytogenes; LCMV: lymphocytic choriomeningitis virus; HSV: herpes simplex virus; VSV: vesicular stomatitis virus; VV: vaccinia virus; Yptb: Yersinia pseudotuberculosis; SI: Small intestine; LP: Lamina propria; IEL: Intraepithelial lymphocytes; FRT: Female reproductive tract; GITRL: glucocorticoid-induced TNFR-related protein ligand; miR: microRNAs.
