# Supplementary material for: Discipline in Stages: Regulating CD8+ Resident Memory T Cells
Source: Front Immunol. 2021 Mar 19;11:624199. doi: 10.3389/fimmu.2020.624199 (PMC8017121; doi:10.3389/fimmu.2020.624199)
Supplement: Supplementary file 3 [file Table_3.pdf]

Supplementary Table 3. Stage 3: CD8$^+$ T$_{RM}$ maintenance in peripheral tissues.

**Stage 3: Maintenance in the tissue**			**Tissue**	**Model**	**References**
TRM markers	CD69	Independent	Lung	Influenza	(99)
Antigen	Independent		Intestinal IEL	LCMV	(104)
			Skin	CPXV	(126)
				DNFB-HSV	(103)
			Lung	Influenza	(99,132)
	Dependent		Lung	Influenza	(128)
Cytokines	IL-15	Survival	Skin	HSV	(93)
	TGF-β	Retention	Intestine	LCMV	(108)
			Skin	HSV	(93)
			Intestinal LP	Yptb	(81)
	IL-12	Survival	Intestinal LP	Yptb	(81)
Transcriptional regulators	Id3↑		Intestinal IEL	LCMV	(90)
	Ahr↑		Skin	HSV - DNFB	(133)
			Intestine	LCMV	(43)
			Lung	Human	(72)
	Notch↑		Lung	Human/Influenza	(72)
Immune mediators	NAD/ P2RX7	Depletion	Intestinal IEL, liver	LCMV	(139)
	IFITM3	Survival	Lung	Influenza	(140)
Metabolism	FABP4/ FABP5		Skin	VV	(141)

			HSV	(142)
	FABP1/FABP4	Liver	LCMV	(142)
	FABP1/FABP2/FABP6	Intestinal IEL	LCMV	(142)
	BTLA, SPRY1, A2AR	Lung	Human/Influenza	(72)
	Tigit	Intestinal IEL	LCMV	(43)

Table abbreviations: ↑ upregulated; DNFB: 2,4-Dinitro-1-fluorobenzene; LCMV: lymphocytic choriomeningitis virus; HSV: herpes simplex virus; VV: vaccinia virus; Yptb: Yersinia pseudotuberculosis; CPXV: cowpox virus; LP: Lamina propria; IEL: Intraepithelial lymphocytes
