# Supplementary material for: Discipline in Stages: Regulating CD8+ Resident Memory T Cells
Source: Front Immunol. 2021 Mar 19;11:624199. doi: 10.3389/fimmu.2020.624199 (PMC8017121; doi:10.3389/fimmu.2020.624199)
Supplement: Supplementary file 4 [file Table_4.pdf]

Supplementary Table 4. Stage 4: Pathogen challenge

**Stage 4: Pathogen challenge**			**Tissue**	**Model**	**References**
Transcriptional regulators	Notch↑	IFN-γ	Lung	Human/Influenza	(72)
	Hobit↑	Granzyme B	Liver	LCMV	(158)
	Bhlhe↑	Metabolism, IFN-γ, GzmB, TNF	Lung	Influenza	(164)
			Intestinal IEL	LCMV	(43)
Immune effector molecules	IFN- γ		Lung	Human/Influenza	(72)
				Influenza	(156)
				Healthy human tissue	(73)
			Skin	HSV	(82,83)
				Human	(24)
			Lung	LM	(92)
				LCMV	(155)
	TNF-α		Lung	Human/Influenza	(72)
	IL-2		Lung	Human/Influenza	(72)
				Healthy human tissue	(73)
			Liver	Human	(157)
	Granzyme B		Intestinal IEL	LCMV	(71)
			Liver	LCMV	(158)
			Brain	LCMV	(155)
			Skin	Human	(24)
	CD49a		Skin	HSV	(24,83)

Immune inhibitors	PD-1	Lung	Human/Influenza	(72)
			Influenza	(128)
	CD101	Intestinal IEL	LCMV	(43)
	CTLA4	Lung	Human/Influenza	(72)
		Intestinal IEL	LCMV	(43)
	LAG3	Lung	Human/Influenza	(72)
		Intestinal IEL	LCMV	(43)
	BTLA, SPRY1, A2AR	Lung	Human/Influenza	(72)
	Tigit	Intestinal IEL	LCMV	(43)

Table abbreviations: ↑ upregulated; LM: listeria monocytogenes; LCMV: lymphocytic choriomeningitis virus; HSV: herpes simplex virus; CPXV: cowpox virus; IEL: Intraepithelial lymphocytes; GzmB: Granzyme B
